# Migration and health right: Probabilistic estimate of the factors that impact on health right of the migrant population, Peru 2019–2021

**DOI:** 10.1371/journal.pone.0288584

**Published:** 2023-12-06

**Authors:** Juan Arroyo-Laguna, Mariella Huánuco, Pedro La Chira, David Jumpa Armas

**Affiliations:** 1 Universidad San Ignacio de Loyola, Lima, Perú; 2 National Superintendency of Health, Intendency for the Promotion of Health Rights, Lima, Peru; 3 Universidad Nacional Federico Villarreal, School of Economy, Lima, Peru; 4 Ministry of Health of Peru, General Directorate of Decentralization, Lima, Peru; National Institute of Public Health: Instituto Nacional de Salud Publica, MEXICO

## Abstract

The study aims to identify factors associated with the violation of the right to health of the regular migrant population with respect to the nonmigrant population in Peru during the period 2019–2021, based on the complaints of health services users. It is a three-year cross-sectional and retrospective study on a total population of 122,505 complainants to the National Superintendency of Health (SUSALUD). The types of health rights used were those established in Peruvian Law No. 29414. An unordered multinomial probability model was used to estimate the probability of belonging to five types of violated rights based on the regular migrant and nonmigrant population, and the exogenous variables that affect this probability. The individual significance tests of the model, the tests for combining categories and the test of independence of irrelevant alternatives by means of the Wald and Hausman-McFadden tests were previously taken. The results indicated an increase in complaints from regular migrants of 5.6% in the 2019–2021 period unlike nonmigrants who had a decrease of 12.2%. The greatest probability that health rights of regular migrants are violated refers to access to information and the right to care and recovery, where their probability of violation is 27.7% and 25.4%, respectively (p-v < 0.05, CI = 95%). Likewise, health rights are more likely to be violated if they are women; if they are adults (41 years old on average); if they do not possess any type of health insurance; if they use Peruvian Ministry of Health (MINSA) services; and if they are located in metropolitan cities, such as Lima and Callao.

## Introduction

Peru has a total population of approximately 33,396,000 people [1]. In the 2017–2021 period, the number of immigrants increased significantly. As of August 2021, the number of foreigners residing in Peru amounted to 1,347,893. According to their nationality, 86.8% are Venezuelans, 3.3% are Colombians, 1.1% are Ecuadorians, 1.0% come from the United States and 1.0% from Spain; the other countries being below 1.0% [2].

In this context of rapid migratory growth, the Peruvian State, in January 2017, created a provisional identity document that would allow migrants to reside and work on the Peruvian soil (Temporary Residency Permit TRP). A migrant in a regular situation is one who has valid documents issued by the Peruvian government authorities, such as the TRP, which allow them to carry out activities within the framework of Peruvian law. However, the central problem was and continues to be the most difficult one: complete integration of the newly arrived people into the new society in which they were going to reside. The literature shows that migrants have been experiencing a difficult labor, social, institutional and cultural insertion and that throughout this process they have often lost rights that they already had in their country of origin or have observed a wide variety of levels and forms of violation or annulment of their rights [3, 4]. Forced displacement, such as that of the Venezuelan population in the last five years, has been dealing with issues of human trafficking, abuse, exploitation, and xenophobia, among others, which have further lead to cultural, family, and social transitions resulting in physical and mental health problems [5]. This study addresses one of these fields of insertion and exclusion: health rights of regular migrants. The literature dedicated to human rights in health and its measurement is vast and has agreements and differences on both topics [6–9]. On one hand, it is very difficult for there to be full agreement on what health rights are, what is the meaning of a “complete physical, mental and social well-being, and not merely the absence of disease or illness” [10]. This brings us to Amartya Sen’s philosophical question, what is a successful life? [11]. The answer varies depending on whether on stars from a consequentialist or deontological, procedural or pragmatic, hedonistic or stoic point of view. A few years ago, Gruskin highlighted the confusion regarding definition of basic issues, such as human rights, ethics, and social justice [12]. However, it is clear that these issues are intertwined.

On the other hand, at the other end of the scale of rights, that of lives and societies with rights that have been nullified or flagrantly violated, there seems to be more agreement, and it is relatively easier to construct indicators that make this absence tangible. The literature has focused on these critical situations as violations of the humanitarian law [13, 14].

Nevertheless, there is a third case, wherein the measurement of human rights is partially exercised or subjected to a structural or chronic violation. There are countries, such as Peru, whose number of preventable diseases has been medium or high for many years. Who draws the limit between the fully exercised right and the violated right and where is this limit? Countries lacking social protection systems, such as universal healthcare, tend to make it difficult, if not impossible, for their population to reach their full potential, particularly in their disadvantaged sectors. Thus impede the full exercise of the human rights to health of their people.

This is the case of Peru, wherein it is implied that the violation of the right to the health of Peruvians, especially of its poor and vulnerable population, is preexisting to the migration boom during 2017–2021. The Peruvian health care system, like that of other Latin American countries, is characterized by high out-of-pocket spending and is fragmented, with different financing and provision schemes according to socioeconomic levels, which generate avoidable inequities [15–17]. Consequently, the negative outcomes in terms of morbidity and mortality reduce the number of years of life of the poorest and most vulnerable people [18, 19].

This health system, with incomplete rights, was obviously not prepared to face the great migratory wave of recent years [20, 21]. According to the technical report sponsored by the IOM on the “Situation of foreign migrants in Peru and their access to social services, health services and education,” between the years 2011 and 2015 when the migrant population was limited and came from average professional layers, 31.2% had private insurance compared to 23.4% who were insured with the Bismarckian fund of Social Health Insurance (ESSALUD), and 11.7% had free public fund of the Integral Health Insurance (SIS) of MINSA [22]. The survey of INEI in 2018, which took place during the massive entry of the migrants, identified that only 8.5% of that population had health insurance, and that 55.1% went to a pharmacies or apothecaries when they had a health problem. Likewise, it revealed that 12.9% suffered from chronic diseases, of which 77.9% did not receive treatment—these being mainly female population [23]. This trend slightly changed in the following year as per the report presented by the Venezuelan Peruvian Business Chamber and Konrad–Adenauer–Stiftung, which identifies that 32.8% of the population surveyed had health insurance; however, within that percentage, 76.7% were affiliated with free public insurance (SIS), 16.5% were enrolled in ESSALUD and 3.9% in private insurance [24]. In summary, the Peruvian case is that of a recipient country with a health system of limited access and coverage, which received a new potential demand of unprotected population.

Moreover, the measurement of health rights is not black and white, which is the reason for the advances and the ongoing debate on the subject. The study objective was to identify factors associated with the violation of the right to health of the regular migrant population with respect to the nonmigrant population in Peru during the period 2019–2021, based on the complaints registered in the National Superintendence of Health (SUSALUD). In that sense, the proposed hypothesis is that migratory status increases the probability of suffering the violation of some of their health rights, as compared to nonmigrants. We have started with the evaluation of the rights of migrants at the peak of the phenomenon, flagrant cases, or cases of open disagreement between patients and health service providers, which oblige migrant users to file a complaint despite the large investment of time involved.

## Methodology

The present study used a probabilistic model for the evaluation of the right to the health of migrants by examining complaints in services in Peru during the period January 2019– September 2021. The study was conducted on anonymous databases made publicly available by SUSALUD, as established by Legislative Decree No. 1158 of December 2013. This registry includes the anonymous complaints of migrants and nonmigrants, allowing comparisons of the characteristics of the rights violated in both groups. The study did not involve humans. There were no interviews or fieldwork with individuals or populations. For the classification of complaints according to types of health rights, we used the regulation of Law No. 29414, “Law that establishes the rights of users of health services” contained in Supreme Decree No. 027-2015-SA. Such regulation differentiates five types of user rights: access to information, access to health services, the right to healthcare and recovery, right to informed consent, and right to protection of rights. Under this classification, the Peruvian Ministry of Health collects complaints from all the Institutions Providers of Health Services (IPRESS) across the country. Table 1 depicts items that comprise each type of right according to the classification established by Law No. 29414.

**Table 1:** Classification of the rights of users of health services according to Law No. 29414.

Right to Access to Health Services	Right of Access to Information	Right to Health Care and Recovery	Right to Informed Consent	Rights Protection
1. To emergency care, without conditioning to the presentation of any document	1. To be properly and timely informed as a user	1. To be attended by health personnel authorized by current regulations	1. To receive informed consent in writing in the following cases:	1. To be heard and receive a response to their complaint or claim by the corresponding instance, when dissatisfied with the care received
2. To the free choice of health professional or IPRESS	2. To determine the name of the doctor responsible for their care, as well as professionals in charge of the procedures	2. To be treated with full respect for their dignity and privacy, as well as kindness and without discrimination	a. In the case of risky tests, surgical interventions, surgical contraception, or procedures that may affect their integrity, except in an emergency	2. To receive immediate treatment and request reparation in the corresponding manner for the damages caused in the IPRESS
3. To receive care with freedom of clinical judgment	3. To receive necessary and sufficient information, with kindness and respect, regarding the conditions for the use of health services, prior to receiving care.	3. To receive scientifically proven treatment or be warned regarding adverse reactions and side effects	b. In the case of exploitation, treatment or display of images for educational purposes	3. To have access to their clinical history and epicrisis
4. To a second medical opinion	4. To receive necessary and sufficient information about their transfer inside or outside the IPRESS, as well as granting or denying their consent, unless justified by the IPRESS representative	4. To their personal safety, not to be disturbed or exposed to danger by people outside IPRESS	c. Before being included in a scientific research study	4. To the confidential nature of the information contained in your clinical history
5. To the access to educated and necessary services, medicines, and health products	5. To receive necessary and sufficient information on the rules, regulations, and/or administrative conditions related to their care from IPRESS	5. To authorize the presence of third-parties during medical examination or surgery, with the consent of the treating physician	d. When they receive application of products or procedures under investigation	
	6. To obtain complete, timely, and continuous information about their own condition and treatment options from their treating physician in easily understandable terms	6. To respect the natural process of their death as a consequence of the terminal state of the disease	e. When they have made the decision to refuse to receive or continue treatment, except when their life or public health is at risk	
	7. To decide their voluntary withdrawal from the IPRESS expressing this decision to the treating doctor		f. When the patient receives palliative care	
	8. To refuse to receive or continue treatment			
	9. Right to be informed about the experimental condition of products or procedures, as well as their risks and side effects			

Source: Law No. 29414, El Peruano [25].

The dependent variables involved in the model were these five types of rights, although one more item was identified in the database as “other,” which was mantained. Migration, sex, health sector, COVID-19 cases, and their subcategories were considered as explanatory variables of interest. The explanatory control variables were age group, macro region, type of channel (telephone, delegates, written, in person, virtual), type of insurance, and period.

Initially, the sociodemographic profile of the complainants and characteristics of the violated rights were reviewed. Second, significant differences between both groups were validated; i.e., migrants and nonmigrants. Third, an unordered multinomial logistic regression was used to rigorously identify factors associated or linked to the violation of health rights in the complaining population, particularly the regular migrant population. In the applied model, it was used an unordered model because of the five types of rights were not ranked and had the same importance. The procedure was the choice of a base category, in our first case "the right to health care and recovery", with respect to which “the factors that determine and/or influence” it were evaluated as dependent variables.

**Fig 1:**
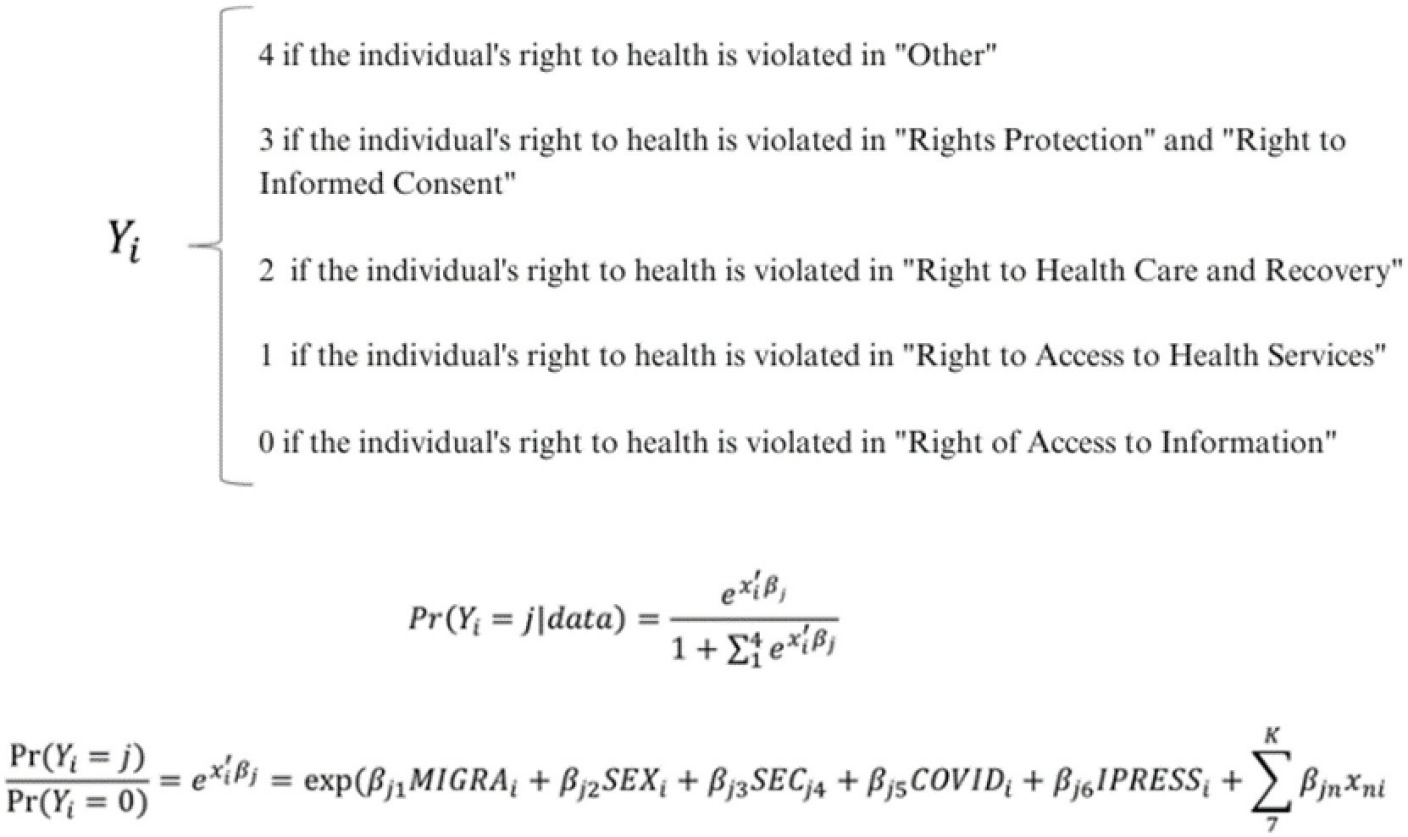
Unordered multinomial logistic regression model.

Considering the foregoing, the model is shown in Fig 1.

In the formula, xi represents the vector of values for the explanatory variables for the complaint of the affected party i; βj is the vector of coefficients of alternative j. The estimation of the model implies the calculation of five equations, i.e., one for each type of health right that was considered as a base.

The hypothesis of the study proposes that migratory status increases the probability of suffering the violation of some of their health rights by migrants, compared to non- migrants. It was assumed that a probable difference in disadvantage of migrants expresses a gap in the exercise of the right to health. This does not imply that nationals have a full exercise of their rights. However, they probably have so in another magnitude and form. The study further sought to explain which variables best explain these differences. It should be noted that the impact on relative probabilities (odds ratios) was sought rather than absolute probabilities, i.e. the approximate changes that the variables can produce. Before processing the analysis of the main results of the study and testing the hypotheses, the significance of the variables proposed in the model was corroborated. This validation was performed using Wald’s statistical test that verified whether the fusion of two variables made the study more representative, which was shown to be unnecessary [26]. Likewise, the Hausman–McFadden statistical test was used to verify the acceptance of the assumption of independence of irrelevant alternatives (IIA, for its acronym) [27]. All tests were evaluated at a significance level of 1%, 5%, and 10%.

Based on the above mentioned factors, an attempt was made to estimate the probability of the violation of a certain group of rights, considering certain exogenous variables. The main attribute of this regression is that it allows the use of unordered categorical variables as dependent variables.

## Results

### Result 1: Complaints according to sociodemographic characteristics

In the first place, the proportion of complaints registered with respect to those patients who were attended was evaluated; complaints were made by both nonmigrants and migrants during the 2019–2021 analysis period. Results revealed an increase of 5.6% in migrant complaints during the study period and a decrease of 12.2% in nationals’ complaints.

The proportion of migrant complaints was 0.8% of total complaints in 2019; in 2021 it was 1.3% of the total. Migrant complaints increased between 2019 and 2020 by 3.4%, and between 2020 and 2021 by 4.8%.

It should be noted that both variations occurred in the new context of the pandemic, in which the face-to-face complaints channel was closed, both at the SUSALUD central office and at the health facilities, where the SUSALUD delegates are located. These delegates are health professionals who remain in Level II and III establishments to deal with complaints in situ while mediating with directors and managers.

When performing homogeneity tests, it was possible to determine that the sociodemographic composition of national and migrant complainants is statistically different for most characteristics in the three years of analysis.

Regarding the profile of the migrant complainant, approximately 50% of registered complaints were migrants between 30 and 59 years of age, followed by 30.9% of complainants between 18 and 29 years of age. Additionally, it was found that most of the migrant complaints came from women (58.5%) and not from men (41.5%), which could express a double discrimination, as migrants and as women. The largest number of migrant complaints was made in metropolitan cities, such as Lima and Callao, with 81.5%, followed by the northern and southern macro regions with 7.5% and 6.1%, respectively. In relation to the provider institutions where the migrants’ complaints originated, 90.3% correspond to complaints that occurred in the health facilities of the MINSA, followed by 4.0% of complaints in ESSALUD. This proportion probably relates to the location of the majority of migrants in informal jobs, as access to ESSALUD requires a formal contract. Finally, analysis suggests that migrants have a higher percentage of access to health facilities outside their own districts of residence, as 57.4% of their complaints were in facilities outside their districts of residence (see Table 2). In relation to the attention channel where the complaints are registered, it shows that both migrants and non-migrants prefer to register their complaints in person rather than using indirect or virtual channels.

**Table 2:** Peru: Complaints from affected migrants and nonmigrants according to sociodemographic characteristics, January 2019-September 2021.

Variables	Total		Migrant		Nonmigrants		Percentage difference	
	Abs.	%	Abs.	%	Abs.	%	P > t	Significance
**Total**	**122 505**	**100**	**1 150**	**100**	**121 355**	**100**		
**Sex**							3,9341	**
Men	54 356	44,4	477	41,5	53 879	44,4		
Women	68 149	55,6	673	58,5	67 476	55,6		
**Age group**							315,41	***
From 0 to 11 years old	14 668	12	123	10,7	14 545	12		
From 12 to 17 years old	4 603	3,8	28	2,4	4 575	3,8		
From 18 to 29 years old	19 063	15,6	355	30,9	18 708	15,4		
From 30 to 59 years old	53 477	43,7	548	47,7	52 929	43,6		
Over 60 years old	30 694	25,1	96	8,3	30 598	25,2		
**Macro region**							188,1499	***
Lima and Callao	77 006	62,9	937	81,5	76 069	62,7		
North Macroregion	11 791	9,6	86	7,5	11 705	9,6		
South Macroregion	14 125	11,5	70	6,1	14 055	11,6		
Central Macroregion	13 771	11,2	44	3,8	13 727	11,3		
East Macroregion	5 812	4,7	13	1,1	5 799	4,8		
**Provider Institution**							84,8312	***
MINSA/GORE	100 761	82,3	1038	90,3	99 723	82,2		
ESSALUD	14 923	12,2	46	4	14 877	12,3		
Private	5 812	4,7	66	5,7	5 746	4,7		
Armed Forces and National Police of Peru	743	0,6	-	-	743	0,6		
Other	300	0,2	-	-	266	0,2		
**Location of the complaint**							223,4745	***
Same district as IPRESS	29 276	23,9	490	42,6	28 786	23,7		
Different district than the IPRESS	93 229	76,1	660	57,4	92 569	76,3		
**Year of presentation of the complaint**							689.070	***
2019	46 352	37.8	354	30.8	45 998	37.9		
2020	44 610	36.4	379	33.0	44 231	36.4		
2021	31 543	25.7	417	36.3	31 126	25.6		
**Support channels before the pandemic**							113,0717	***
Telephone	25 232	20,6	158	13,7	25 074	20,7		
Delegates	28 329	23,1	386	33,6	27 943	23		
Written	2 155	1,8	29	2,5	2 126	1,8		
In person	51 842	42,3	397	34,5	51 445	42,4		
Virtual	14 947	12,2	180	15,7	14 767	12,2		

*/ Significant difference (p < 0.10).

**/ Highly significant difference (p < 0.05).

***/ Very highly significant difference (p < 0.01).

Source: Author’s own elaboration based on SUSALUD—SADERECHOS—IPROT—Base of complaints, 2019–21.

### Result 2: Migrant and nonmigrant complaints before and during the pandemic

In terms of the categories of health rights that were violated in the migrant and nonmigrant populations, the most common category of right was access to healthcare, which accounted for 12.5% of complaints before the pandemic and 25.5% during the pandemic. Regarding these complaints, it is evident that during the COVID-19 pandemic, access to health services was more difficult owing to the overload of the health system. It is also observed that complaints doubled due to lack of access to information (from 7.2% to 14%) and those related to healthcare and recovery increased (from 8.3% to 12.3%) (see Table 3).

**Table 3:** Peru: Complaints of affected migrants and nonmigrants according to the types of health rights and cases related to COVID-19, January 2019-September 2021.

Variables	Total		Migrant		Nonmigrants		Percentage difference	
	Abs.	%	Abs.	%	Abs.	%	P > t	Significance
**Total**	**122 505**	**100**	**1 150**	**100**	**121 355**	**100**		
**Sex**							3,9341	**
Men	54 356	44,4	477	41,5	53 879	44,4		
Women	68 149	55,6	673	58,5	67 476	55,6		
**Age group**							315,41	***
From 0 to 11 years old	14 668	12	123	10,7	14 545	12		
From 12 to 17 years old	4 603	3,8	28	2,4	4 575	3,8		
From 18 to 29 years old	19 063	15,6	355	30,9	18 708	15,4		
From 30 to 59 years old	53 477	43,7	548	47,7	52 929	43,6		
Over 60 years old	30 694	25,1	96	8,3	30 598	25,2		
**Macro region**							188,1499	***
Lima and Callao	77 006	62,9	937	81,5	76 069	62,7		
North Macroregion	11 791	9,6	86	7,5	11 705	9,6		
South Macroregion	14 125	11,5	70	6,1	14 055	11,6		
Central Macroregion	13 771	11,2	44	3,8	13 727	11,3		
East Macroregion	5 812	4,7	13	1,1	5 799	4,8		
**Provider Institution**							84,8312	***
MINSA/GORE	100 761	82,3	1038	90,3	99 723	82,2		
ESSALUD	14 923	12,2	46	4	14 877	12,3		
Private	5 812	4,7	66	5,7	5 746	4,7		
Armed Forces and National Police of Peru	743	0,6	-	-	743	0,6		
Other	300	0,2	-	-	266	0,2		
**Location of the complaint**							223,4745	***
Same district as IPRESS	29 276	23,9	490	42,6	28 786	23,7		
Different district than the IPRESS	93 229	76,1	660	57,4	92 569	76,3		
**Year of presentation of the complaint**							689.070	***
2019	46 352	37.8	354	30.8	45 998	37.9		
2020	44 610	36.4	379	33.0	44 231	36.4		
2021	31 543	25.7	417	36.3	31 126	25.6		
**Support channels before the pandemic**							113,0717	***
Telephone	25 232	20,6	158	13,7	25 074	20,7		
Delegates	28 329	23,1	386	33,6	27 943	23		
Written	2 155	1,8	29	2,5	2 126	1,8		
In person	51 842	42,3	397	34,5	51 445	42,4		
Virtual	14 947	12,2	180	15,7	14 767	12,2		
**Before Pandemic**	**46 352**	**37.8**	**354**	**30.8**	**45 998**	**37.9**	**191.700**	***
Access to Information	8 835	7.2	70	6.1	8 765	7.2		
Access to Health Services (ICU beds, emergency)	15 295	12.5	87	7.6	15 208	12.5		
Health Care and Recovery	10 216	8.3	80	7.0	10 136	8.4		
Protection and Informed Consent	4 793	3.9	37	3.2	4 756	3.9		
Others	7 213	5.9	80	7.0	7 133	5.9		
**During pandemic**	**76 153**	**62.2**	**796**	**69.2**	**75 357**	**62.1**	**73.359**	***
Access to Information	17 181	14.0	201	17.5	16 980	14.0		
Access to Health Services	31 254	25.5	330	28.7	30 924	25.5		
Health Care and Recovery	15 002	12.2	142	12.3	14 860	12.2		
Protection and Informed Consent	6 419	5.2	58	5.0	6 361	5.2		
Others	6 297	5.1	65	5.7	6 232	5.1		
**No COVID-19**	**58 647**	**47.9**	**645**	**56.1**	**58 002**	**47.8**	**247.844**	***
**COVID-19**	**17 506**	**14.3**	**151**	**13.1**	**17 355**	**14.3**	**145.355**	**0.150**

*/ Significant difference (p < 0.10).

**/ Highly significant difference (p < 0.05).

***/ Very highly significant difference (p < 0.01).

Source: Author’s own elaboration based on SUSALUD—SADERECHOS—IPROT—Base of complaints, 2019–21.

In relation to complaints not related to COVID-19, some significant differences can be observed between migrants and nonmigrants. In majority of cases, migrants complained significantly about elements related to the right to access and the right to care and recovery. During COVID-19, the proportion of migrants and nonmigrants who reported incidents was similar. The main reasons for complaints were rule-out tests, access to information on the patient’s health status, and emergency access to a health facility. However, complaints of both groups were not significantly different, i.e., the same facts were proportionally reported, except for the subtype referring to access to ICU beds, in which migrants presented less than half of the complaints of nationals. This withdrawal of migrants as complainants during the health crisis on this issue requires a specific study. The data only reflects migrants’ perceptions of a lengthy wait that is difficult to resolve with a complaint, especially when relatives and friends are unable to obtain ICU beds.

### Result 3: Unordered logistic multinomial model

In this stage, we sought to identify the factors associated with the complainant and their relationship with the probability of violation of a specific health right.

Prior to the corresponding analysis, as explained in the methodology section, the individual significance test of the proposed model variables was performed. The results of the Wald statistical test for independent variables were highly significant (in 11 of 13 cases p < 0.001). Likewise, the possibility of obtaining more efficient estimators by merging alternatives into a single category was analyzed. The results of the Wald test to combine categories revealed that the hypothesis of no distinction was rejected for each pair of categories of the dependent variable (10 of 10 fusions resulted in p < 0.01), i.e., the fusion of the mentioned categories was inconvenient. Finally, the Hausman–McFadden statistical test was used to corroborate the assumption of IIA, which assumes that the odds ratio between two alternatives does not depend on other categories. The results demonstrate that each of the categories or types of health rights is irrelevant for the calculation of the probability ratios that do not involve it, i.e., we verified the difference between the estimators obtained using all the categories and omitting one if it is significant, and the results show that the IIA assumption has not been violated (for the five types of rights, p > chi square).

Once the suitability of the proposed model was corroborated, the unordered logistic multinomial model was estimated (see Table 4).

**Table 4:** Peru: Results of the effects per migrant and nonmigrant according to of health right and sex.

**Types of health rights**	**Probability**	
Access to information	21.08%	
Access to health services	39.36%	
Healthcare and recovery	21.56%	
Protection of rights and informed consent	7.74%	
Other	10.26%	
**Types of health rights**	**Migrant**	**Nonmigrant**
Access to information	27.76%	25.24%
Access to health services	40.90%	44.07%
Healthcare and recovery	21.21%	20.56%
Protection of rights and informed consent	3.08%	3.90%
Other	7.03%	6.21%
**Sex**	**Migrant**	**Nonmigrant**
**Women**		
Access to information	45.92%	43.18%
Access to health services	20.91%	19.46%
Healthcare and recovery	12.54%	15.67%
Protection of rights and informed consent	3.07%	6.72%
Other	17.53%	14.95%
**Men**		
Access to information	44.60%	41.99%
Access to health services	20.34%	18.96%
Healthcare and recovery	12.92%	16.16%
Protection of rights and informed consent	2.98%	6.52%
Other	19.14%	16.34%

Model adjusted according to control variables: sex, age group, sector and year of filing the complaint

Source: Author’s own elaboration based on SUSALUD—SADERECHOS—IPROT—Base of complaints, 2019–21.

The first result indicates that the types of health rights most likely to be violated are access to health services (39.36% probability), the right to healthcare and recovery (21.56% probability), and right to access information (probability of 21.08%). The other types of rights are far from the first three.

The second result indicates that, in general, migrants are more likely to have each of the types of health rights violated than nonmigrants. Additionally, migrants are more likely to have their health rights violated than nonmigrants in terms of access to information (27.76% vs. 25.24%), healthcare and recovery (21.21% vs. 20.56%), and others (7.03% vs. 6.21%) (see Table 4). However, in terms of the right to protection of rights and the right to informed consent, probabilities of violation for migrants are lower than for nationals.

The third result indicates that the probability of immigrants’ health rights being violated relative to non-immigrants increases if they are women. In relation to age, if it is about young people and adults, the probability increases by 0.24% points in terms of violation of the right to protection of rights, confidentiality, and informed consent. For people with no insurance, the probability increases by 1.53% points in terms of the violation of health services. If they receive attention in the MINSA, the probability increases by 3.19% points in terms of the violation of access to information. Moreover, if they reside in Lima and Callao, the probability increases by 2.45% points in terms of the violation of access to information.

The fourth result indicates that, in general, migrant women are more likely to have their rights violated compared to migrant men. They have higher odds on three of the five types of rights. When comparing the chances of migrant women and men experiencing rights violations, migrant women have a higher chance of observing their rights being violated than migrant men.

The fifth result shows that, when comparing migrant women with nonmigrant women, migrant women are more likely to witness their rights being violated in terms of access to information (45.92% vs. 43.18%) and access to health services (20.91% vs. 19.46%). However, they show a lower propensity for violation of the protection of rights and informed consent (3.07% vs. 6.72% in the national ones).

In sixth place, it is observed that, in the case of male complainants, health rights that are most likely to be violated are the right to access information and right to access health services (44.6% and 20.3%, respectively, compared to 42.0% and 19.0%, respectively, in nonmigrants). They are also less likely to have their right to protection of rights and right to informed consent violated (2.9%) than a nonmigrant.

## Discussion

The purpose of the study was to identify factors associated with the violation of the right to health of regular migrants, which increase the risk of its violation with respect to the nonmigrant population in Peru, based on the data gathered during the period 2019–2021. The study has led to the identification of the variables that make the regular migrant population more prone to the violation of their rights in Peru, as well as an approximation to the probability of migrants’ health rights being violated through the analysis of databases of complaints from health service users.

With respect to complaints, the results revealed an increase of 5.6% among migrants and a decline of 12.2% among nonmigrants, during the period 2019–2021. The COVID-19 pandemic probably modified the behavior pattern of users who were not satisfied with the services. Some studies on user satisfaction during the pandemic showed a notable increase in nonconformity [28–30]. It was determined that the main right violated in the migrant population was access to health services, which before the pandemic represented 7.6% of complaints, increasing to 28.7% during the pandemic. The distribution of complaints among migrants was greater among women (58.5%) and among migrants between 30 and 59 years of age (47.7%). Also, 81.5% of the complaints were located in Metropolitan Lima–Callao, and 90.3% of the complaints were filed against MINSA facilities, which has to do with the informal work of the majority of migrants, who do not have access to ESSALUD, which requires a formal labor regime. The results match with the proposed hypothesis that migrant rights are more likely to be violated than nonmigrant rights, and that there are varied probabilities of violation depending on the type of health right. The study was able to explain which variables best explain these differences. The study’s originality lies in the fact that it has a certain predictive character as it is probabilistic, which must serve the integration process of migrants and the improvement of access and care and recovery of health services in Peru for them.

Two debates emerge in the context of the existing literature, one of a methodological nature and the other of a substantive nature or about results; both debates are connected. The methodological discussion deals with the type of population studied to appreciate the right to health of migrants, the complainants. The complainant is a fraction of the dissatisfied—the dissatisfied user who goes to the legal formalization of their dissatisfaction considering it as serious. In Hirschman’s classic study on Exit, Voice, and Loyalty, the author highlighted the difference between those individuals who complain and stay and those who complain and “leave the lending institution” [31]. In this context of open controversy, the complaint judicializes the protest. The violation of the right must have been more evident to the perception of the user. We have selected this strip because in these cases the measurement of human rights and the right to health appears transparent; however, it is not the only case, as we anticipated.

It is also true that the proportion between the dissatisfied and complainants varies according to the countries and this makes the results variable and not very comparable. There are countries with greater or lesser enforcement of rights, but also populations with greater or lesser sensitivity and resilience. In the study of Gal and Doron, in Israel, based on a telephone survey of 1,500 people, 25% had complaints, but only 9.5% expressed it, and 17% of the latter formalized it based on the law [32]. In contrast to our results, minority groups and recent immigrants had significantly lower rates of grievances and actual complaints. In a 12-month study conducted in the United States, Murad, Gjerde, Bobula, Ostrov, and Murad sought to determine whether gender of patients or providers was associated with complaints; the odds ratio for female patients was associated with complaints (3.10 with a confidence interval of 95%: 1.73–5.55; P < 0,001) [33]. This result is consistent with the results of our study wherein migrant women are more prone than men to complaints. Ngongo, Carlier, and Moles examined the frequency and main complaints of patients attending the emergency department of a university hospital in Belgium, in a seven-year retrospective study, in which they analyzed 155 main complaints from a total of 496,816 patients who attended the emergency department in that period. Complaint rate was 3.1 per 10,000 visits [34]. In Singapore, Lim, Tan, Goh, and Ling investigated 226 complaints involving 5,620,834 attendees over the course of two years, yielding a complaint rate of 4 per 100,000 attendees per year. Approximately 64% of the complaints were verbal and the rest were in writing [35].

In other words, dissatisfaction is higher than complaints and complaints are in most cases low, with the rate of complaints being even lower, but all cases are different. In these cases, the complaints of migrants and nonmigrants were not examined. In the case of Peru, during the period 2019–2021, 611,558 migrants were served, with a total of 1,150 complaints, resulting in a rate of 1.88 per 1,000 served [36]. The levels of dissatisfaction in each country and rates of formal complaints appear to be linked to the level of access and quality of services and resilience of users.

Alternatively, reasons for protests and complaints are highly variable by country and within countries, according to layers and social groups or other variables. For Otarola, vulnerable consumers place more importance on their relationship or trust with their doctor [37]. Mendoza, Armbrister and Abraído-Lanza conducted a study on 419 female Latin American immigrants in the United States and found that perceived social mobility of the migrants was the key to determine which patients complained [38]. Mangrio and Sjögren Forss conducted a systematic review of the literature on the experience of refugees with health systems of the receiving countries, determining that communication, support, and intercultural adaptation of services were the most valued by them [39]. In the study conducted by Ngongo, Carlier, and Mols, complaints were related to lack of communication (39.0%), long waiting times (14.0%), misdiagnosis (22.0%), and poor treatment (13,0%) [34]. In the study conducted by Lim, Tan, Goh, and Ling, the main reasons for complaint were related to attitude/behavior (28.8%), professional skills (17.8%), patient expectations (16.2%), waiting time (10.0%) and communication (7.8%) [35]. There were no particular differences of sex or ethnic group, variables that differ with our results, which highlight gender and national origin.

The complainants are in most cases, then, the apex of the broader problem of violations of rights in terms of quality and access, and as such, they provide clues for the improvement of services. Therefore, this probabilistic methodology of migrant and nonmigrant entitlements adds to the forms of measurement of health rights in use. Literature on health rights monitoring has not focused on this area of serious confrontation, which has been applied more to cases of refugees or humanitarian law. Rather, attempts have occasionally been made to apply categorical variables (yes/no) to estimate the violation of rights in health systems that express incomplete rights or limited rights. Here is a new source for future research and evidence for public health policies. Also, the study opens up the need for in-depth research on migrants’ complaints.

### Study limitations

The study has certain limitations. One of them is that it does not examine unreported violations or all the factors that affect the health rights of migrants and nonmigrants comparatively.

Another limitation is that it only collects complaints from migrants with immigration documents; the complaints procedure in Peru implies identification with a passport, immigration card, work permit, or temporary residence permit, for which the study covers the migrant population that is documented.

Finally, in the years prior to the study period, complaints were registered with another classification of categories: queries, requests for intervention, and complaints. This precludes the comparison of the years 2019–2021 of the present study with the previous years. It was observed that between the years 2012 and 2016, the manifestations of discomfort among users of health services increased: consultations with SUSALUD increased in that period from 1,031 to 30,822; requests for intervention increased from 7,307 to 65,887; and complaints increased from 301 to 1,592 [40].
